# Supplementary material for: Structural insights into the regulation of monomeric and dimeric apelin receptor
Source: Nat Commun. 2025 Jan 2;16:310. doi: 10.1038/s41467-024-55555-6 (PMC11697037; doi:10.1038/s41467-024-55555-6)

Supplementary Information

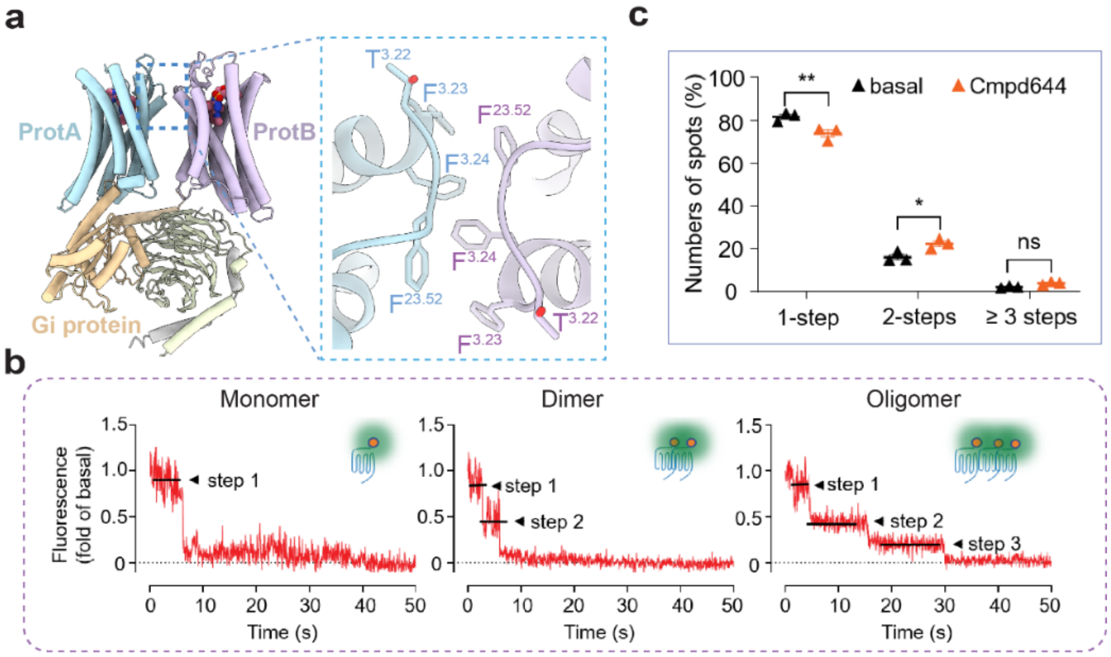

Supplementary Figure 1. Characterization of APJR dimerization on cell surface by the single-molecule imaging approach. a, APJR dimer and downstream Gi-protein complex surface model from cmpd644-APJR-Gi complex (PDB ID: 7W0L). The dimer interface "FGTFF" motif is shown in sticks. **b,** Representative fluorescence time course for an individual molecule. Left, 1-step photobleaching (monomer); middle, 2-steps photobleaching (dimer) and right, 3-steps photobleaching (trimer). **c,** Histogram of photobleaching step numbers for all Snap-APJR molecules analyzed. 1867 spots from 9 movies in basal group (no-treatment); and 1234 spots from 8 movies in cmpd644 group (1 mm treatment for 30 min) were analyzed. Data are mean ± SEM from three biologically independent experiments and were analyzed using two-way ANOVA with Tukey's multiple comparisons test. Source data are provided as a Source Data file.

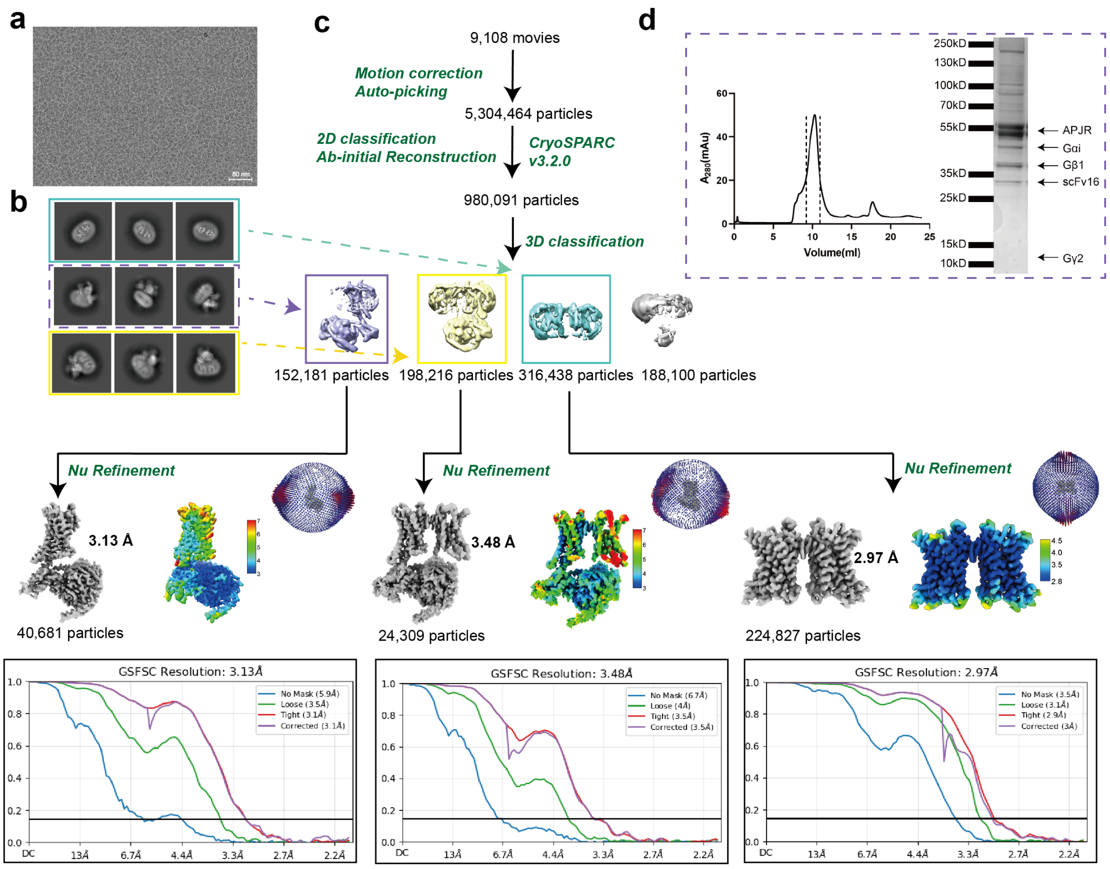

Supplementary Figure 2. Cryo-EM sample preparation, data collection and processing for APJR^{AP13}-Gi-scFv16 complex and apo-APJR. a-d, Representative cryo-EM image derived from 9,108 micrographs of the APJR^{AP13}-Gi-scFv16 complex, with a scale bar of 50 nm (**a**). Representative 2D averages showing distinct secondary structural features from various perspectives of the complex (**b**). Cryo-EM data processing workflow: The data underwent processing using CryoSPARC, and density map images were generated in UCSF Chimera. The final 3D density maps are color-coded based on local resolution and angular distribution of the particles used for the final reconstruction. Gold-standard FSC curves from Phenix indicate nominal resolutions of 3.48 Å, 3.13 Å, and 2.97 Å, employing the FSC = 0.143 criterion for the $_{dim}$APJR^{AP13}-Gi map, $_{mon}$APJR^{AP13}-Gi map, and apo-APJR, respectively (**c**). Elution profile and gel image of APJR (comprising residues 1–350), Gαi, Gβ, Gγ, and scFv16 after SEC purification. The collected fraction for cryo-EM sample were marked between dashed lines (**d**).

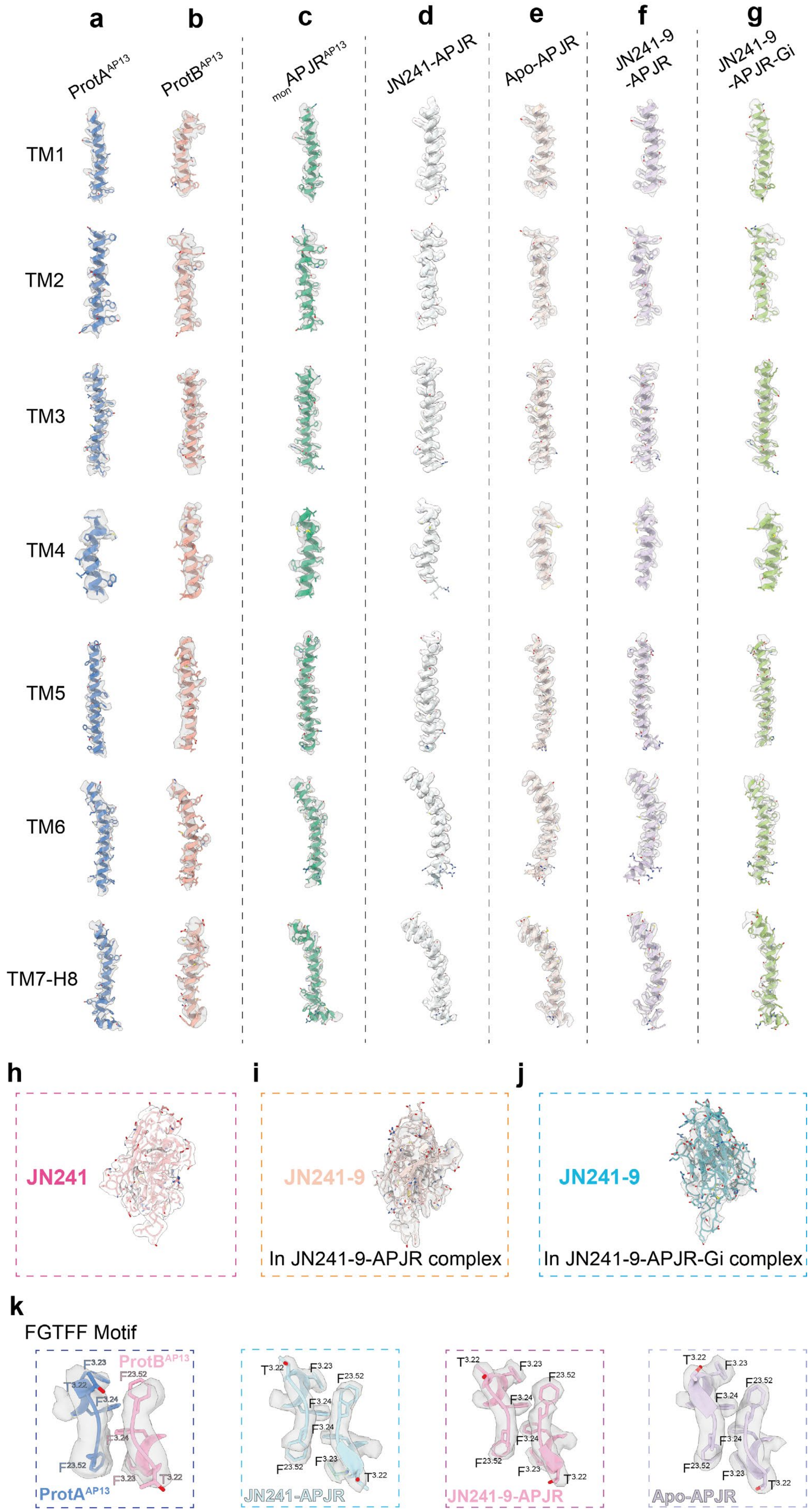

a
b
c
d
e
f
g
ProtA^AP13
ProtB^AP13
monAPJR^AP13
JN241-APJR
Apo-APJR
JN241-9 -APJR
JN241-9 -APJR-Gi
TM1
TM2
TM3
TM4
TM5
TM6
TM7-H8
h
i
j
JN241
JN241-9
In JN241-9-APJR complex
JN241-9
In JN241-9-APJR-Gi complex
k
FGTFF Motif
ProtA^AP13
ProtB^AP13
JN241-APJR
JN241-9-APJR
Apo-APJR

Supplementary Figure 3. Cryo-EM density maps and models of representative helices from the $_{dim}$APJR^{AP13}–Gi, $_{mon}$APJR ^{AP13}-Gi, JN241-APJR, apo-APJR, JN241-9-APJR and JN241-9-APJR-Gi structures. a-g, Cryo-EM density maps and models of TM1, TM2, TM3, TM4, TM5, TM6, TM7-H8 in the PortA (**a**) and ProtB (**b**) of $_{dim}$APJR^{AP13}–Gi, $_{mon}$APJR ^{AP13}-Gi (**c**), JN241-APJR (**d**), apo-APJR (**e**), JN241-9-APJR (**f**) and JN241-9-APJR-Gi (**g**). **h-j,** Cryo-EM density maps and models of JN241 in JN241-APJR complex (**h**), JN241-9 in the JN241-9-APJR complex (**i**) and JN241-9 in the JN241-9-APJR-Gi complex (**j**). **k,** Cryo-EM density maps and models of FGTFF motif (dimer interface) in $_{dim}$APJR^{AP13}–Gi, JN241-APJR, JN241-9-APJR and apo-APJR complexes, respectively.

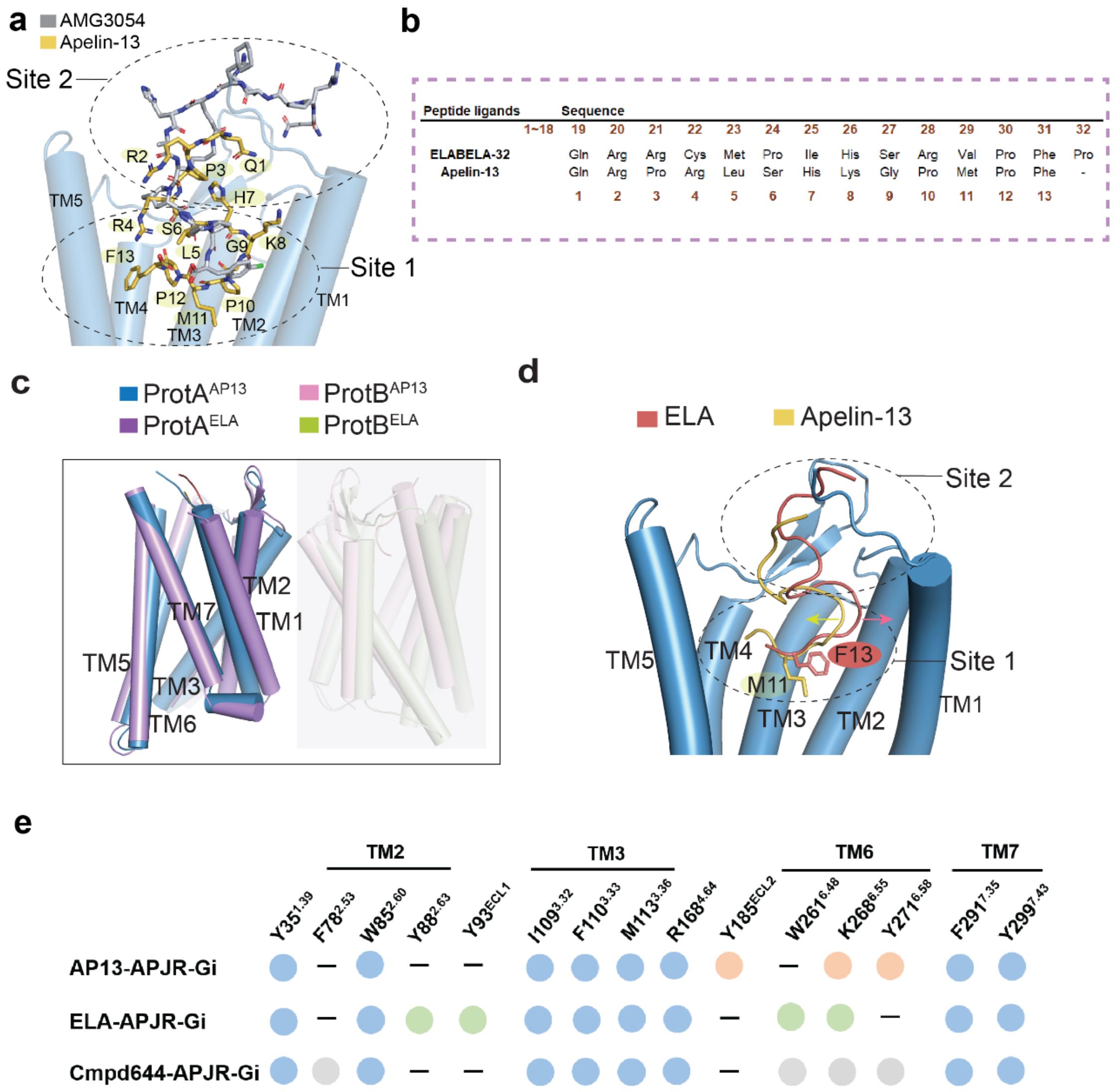

Supplementary Figure 4. Structural comparison between $_{dim}$APJR^{AP13}-Gi and $_{mon}$APJR^{AP13}-Gi/AMG3054-APJR complexes and peptide sequence alignment between apelin-13 and ELA. a, Structural comparison of apelin-13 and AMG3054. Apelin-13 is depicted in yellow, and AMG3054 is depicted in gray. APJR in the ProtA^{AP13} structure is illustrated in blue cylinders, with site 1 and site 2 encircled by dashed lines. **b,** Sequence alignment of ELA-32 and apelin-13. Peptide numbering (upper panel for ELA-32, lower panel for apelin-13) is highlighted in brown. The 1-18 residues of ELA-32 have been omitted. **c,** Structural superposition of ProtAs in apelin-13- with ELA-bound APJR-Gi structure. ProtA^{AP13} and ProtA^{ELA} are colored in blue and purple cylinders, respectively. **d,** Structural comparison of apelin-13 and ELA. Apelin-13 and ELA are shown in yellow and red, respectively, with APJR depicted in blue cylinders. The orientation of the two peptide ligands is indicated with arrows. **e,**

Sequence alignment of key residues of APJR in the ligand binding pocket. The key residues in the ligand-binding pocket of APJR are aligned, with residues interacting with apelin-13 colored in orange, residues interacting with ELA colored in green, and residues interacting with cmpd644 colored in gray. Conserved residues are highlighted in blue.

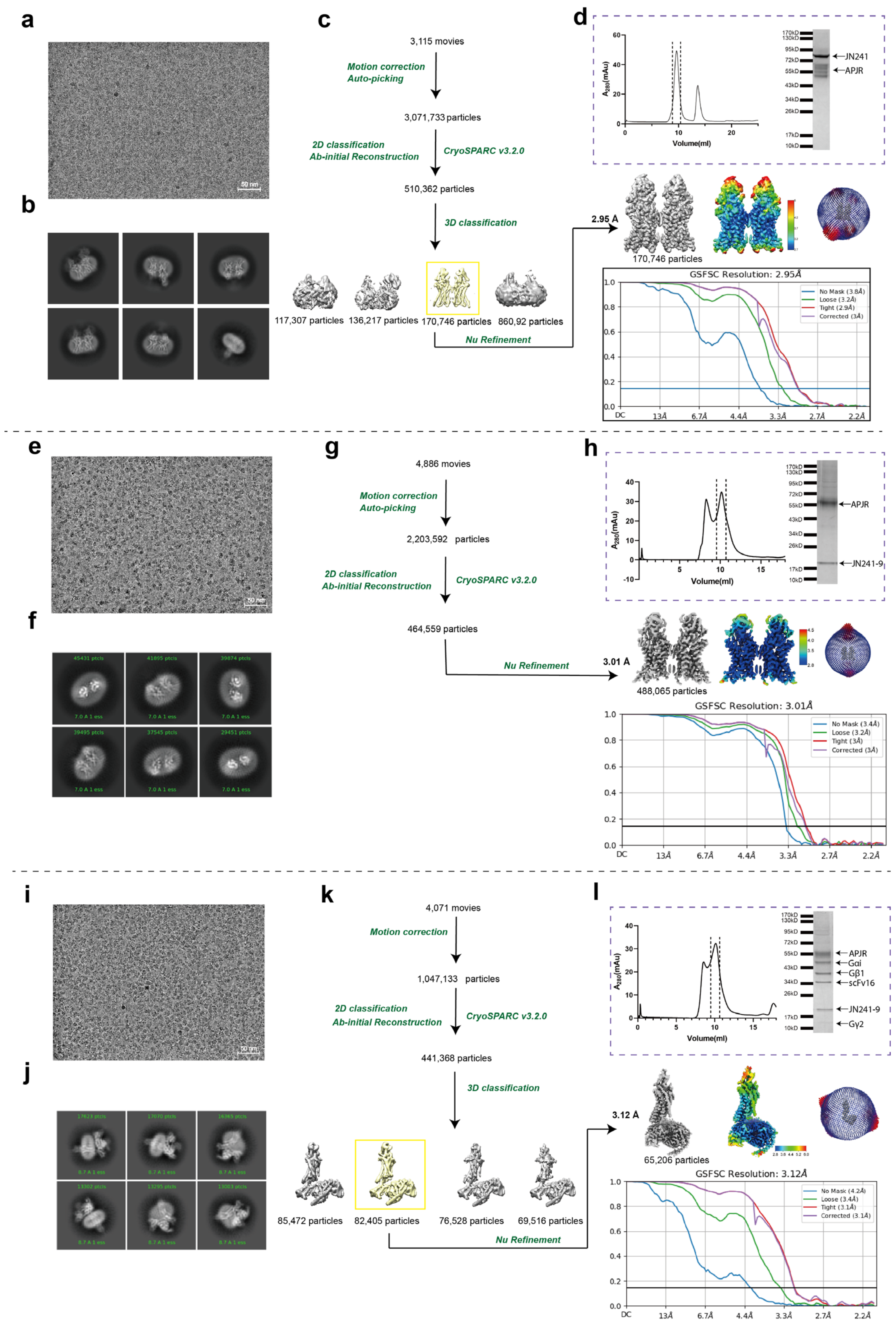

Supplementary Figure 5. Cryo-EM sample preparation, data collection and processing for JN241-APJR. a-d, Representative cryo-EM image derived from 3,115

micrographs of the JN241-APJR complex (50 nm scale) (**a**). Representative 2D averages of complex (**b**). Cryo-EM workflow with CryoSPARC and UCSF Chimera, 3D density maps color-coded by local resolution, angular distribution of the particles used for the final reconstruction, and FSC curves show 2.95 Å resolution (**c**). Elution profile and gel image of APJR and JN241 after SEC purification. The collected fraction for cryo-EM sample were marked between dashed lines (**d**). **e-h,** Representative cryo-EM image derived from 4,886 micrographs of the JN241-9-APJR complex (50 nm scale) (**e**). Representative 2D averages of complex (**f**). Cryo-EM workflow with CryoSPARC and UCSF Chimera, 3D density maps color-coded by local resolution, angular distribution of the particles used for the final reconstruction, and FSC curves show 3.01 Å resolution (**g**). Elution profile and gel image of APJR and JN241-9 after SEC purification. The collected fraction for cryo-EM sample were marked between dashed lines (**h**). **i-l,** Representative cryo-EM image derived from 4,071 micrographs of the JN241-9-APJR-Gi complex (50 nm scale) (**i**). Representative 2D averages of complex (**j**). Cryo-EM workflow with CryoSPARC and UCSF Chimera, 3D density maps color-coded by local resolution, angular distribution of the particles used for the final reconstruction, and FSC curves show 3.12 Å resolution (**k**). Elution profile and gel image of APJR, Gαi, Gβ, Gγ, scFv16 and JN241-9 after SEC purification. The collected fraction for cryo-EM sample were marked between dashed lines (**l**).

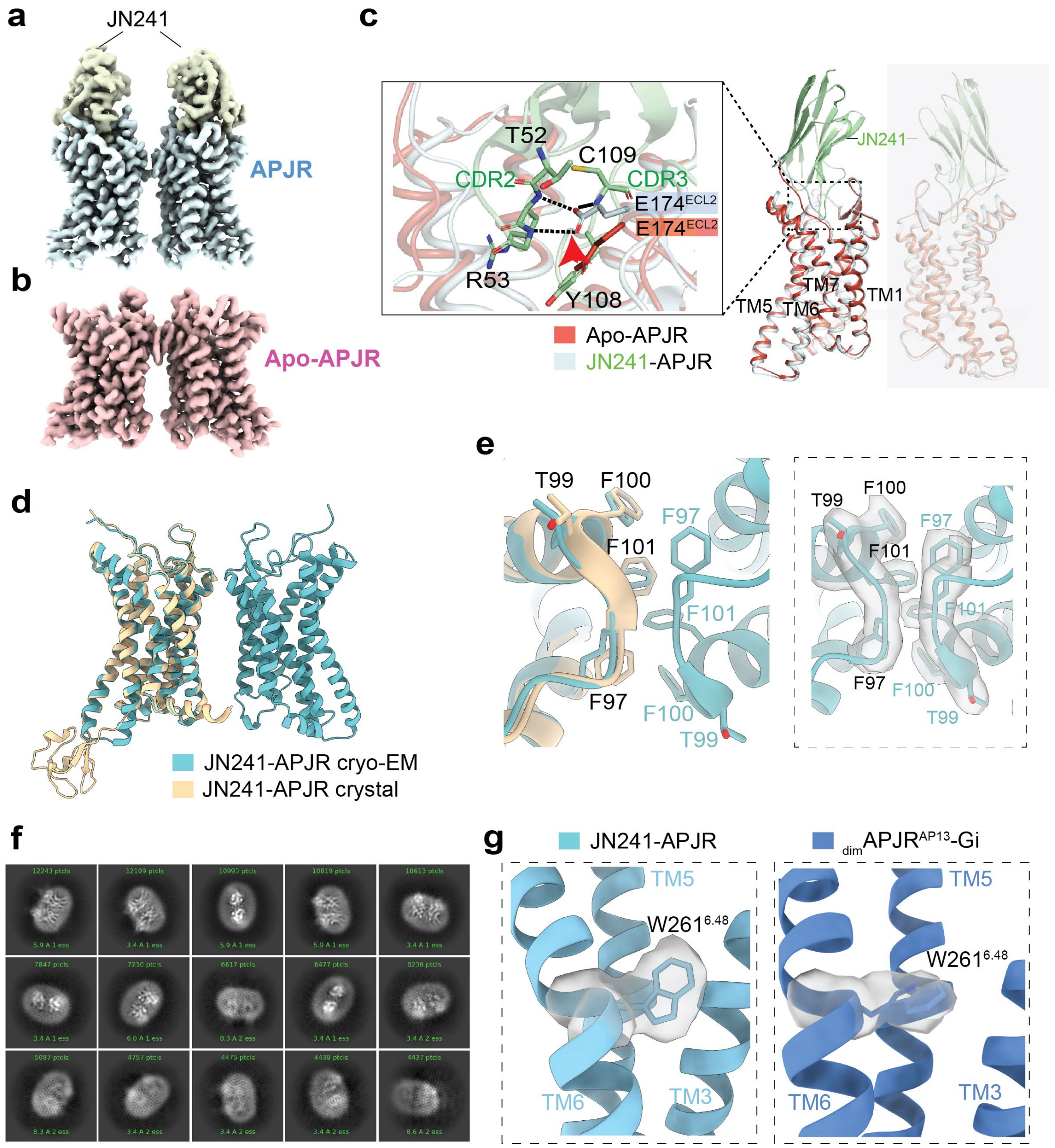

Supplementary Figure 6. Structural characterization of JN241-APJR and apo-APJR dimers, and comparison with ProtB in apelin-13-bound APJR-Gi structure. **a,** Cryo-EM map of JN241-APJR complex. APJR is depicted in light cyan, and JN241 is shown in light green. **b,** Cryo-EM map of apo-APJR without G-protein coupling. Apo-APJR, separated from the dataset of the $APJR^{AP13}$-Gi complex, is colored in pink. **c,** Conformational comparison of $E174^{ECL2}$ in JN241- and apo- APJR. Hydrogen bonds formed by residues T52/R53 from CDR2 of JN241 or residue C109 from CDR3 in JN241 are indicated with arrows. The symmetric partner in the JN241-APJR dimer structure is shaded in gray. **d, e,** Structural comparison between JN241-APJR cryo-EM

and crystal (PDB ID: 6KNM) structures. Overall structural comparison between JN241-APJR cryo-EM and crystal structures (**d**). Superimposition of the crystal structure of JN241-APJR onto cryo-EM structure, with a focus on the dimeric interface of FGTFF motif, shows subtle conformational changes in the side chains of three critical phenylalanine residues. Additionally, the cryo-EM density map for the amino acids comprising the FGTFF motif is shown (**e**). **f,** Representative 2D averages of the JN241-APJR complex, devoid of the Fc tag, consistently exhibit dimer formation, indicating that the presence or absence of the Fc tag does not affect the dimerization of APJR. **g,** Cryo-EM density maps for the "toggle switch" residue $W261^{6.48}$ within the JN241-APJR complex and $_{dim}APJR^{AP13}$-Gi complex structures, respectively.

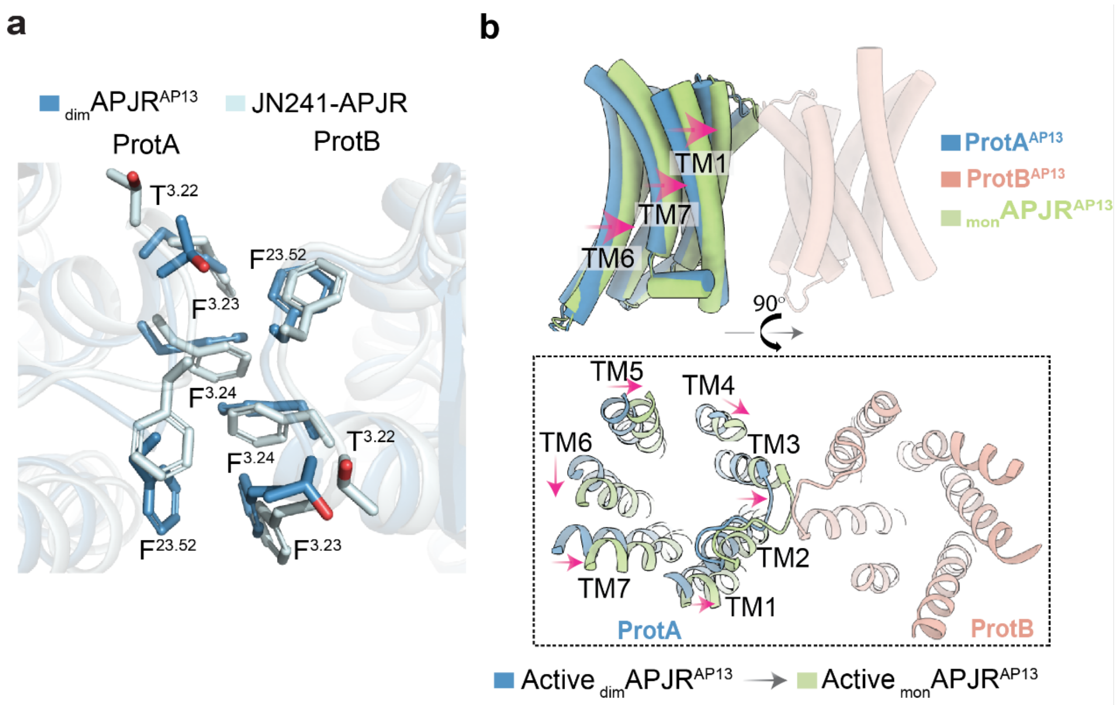

Supplementary Figure 7. Structural comparisons of the dimer interface and the structural transitions from APJR dimer to monomer. a, The "FGTFF motif" residues (shown in sticks) at the dimer interface in apelin-13-bound and antagonist-bound structures are illustrated. **b,** Structural transitions from active dimer to active monomer induced by apelin-13 are depicted. Superposition of $_{dim}$APJR^{AP13}-Gi (ProtA: blue, ProtB: pink) and $_{mon}$APJR^{AP13}-Gi (green) aligned on the Gi protein portion, with the Gi protein omitted.

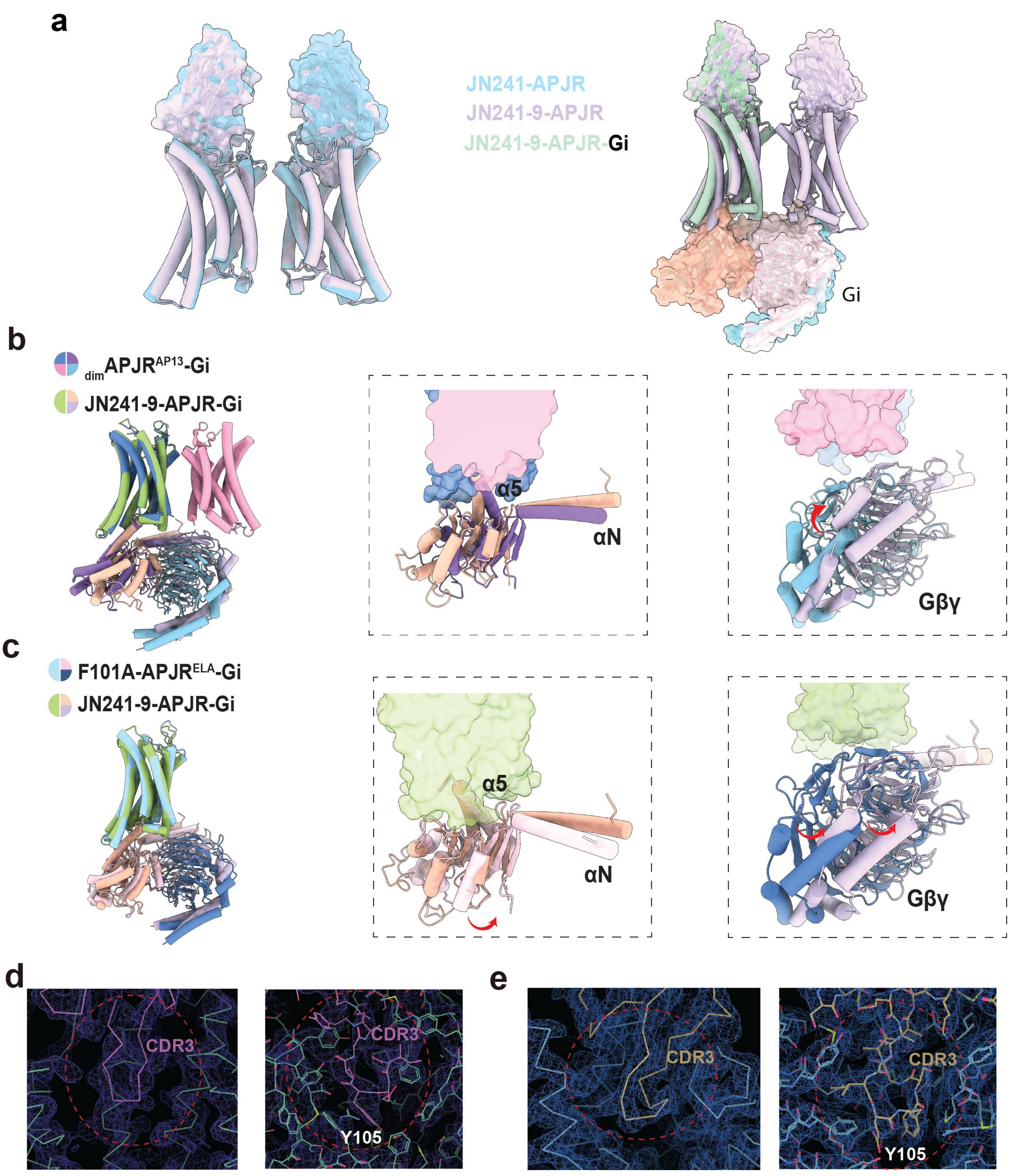

Supplementary Figure 8. The dimerization modes and conformational changes in transmembrane domains of APJR in different functional states. a, Structural alignments of JN241-APJR, JN241-9-APJR, and JN241-9-APJR-Gi complexes. **b,** Structural superposition of $_{dim}$APJR^{AP13}-Gi and JN241-9-APJR-Gi complexes shows conformational shifts in Gα and Gβγ, indicated by arrows. These shifts suggest an upward displacement in the JN241-9-APJR-Gi complex towards the region that would otherwise be occupied by ProtB in the $_{dim}$APJR^{AP13}-Gi complex. **c,** Structural superposition of F101A-APJR^{ELA}-Gi (PDB ID: 7W0P) and JN241-9-APJR-Gi

complexes. The conformational changes of Gα and Gβγ were indicated as arrows. **d, e,** Cryo-EM maps of CDR3 region of JN241-9 in the orthosteric pocket, with the key residue Y105 highlighted: (**d**) CDR3 region of JN241-9 in the Gi protein-bound structure, shown as cartoon (left) and sticks (right), respectively; (**e**) CDR3 region of JN241-9 in the Gi protein-free structure, shown as cartoon (left) and sticks (right), respectively.

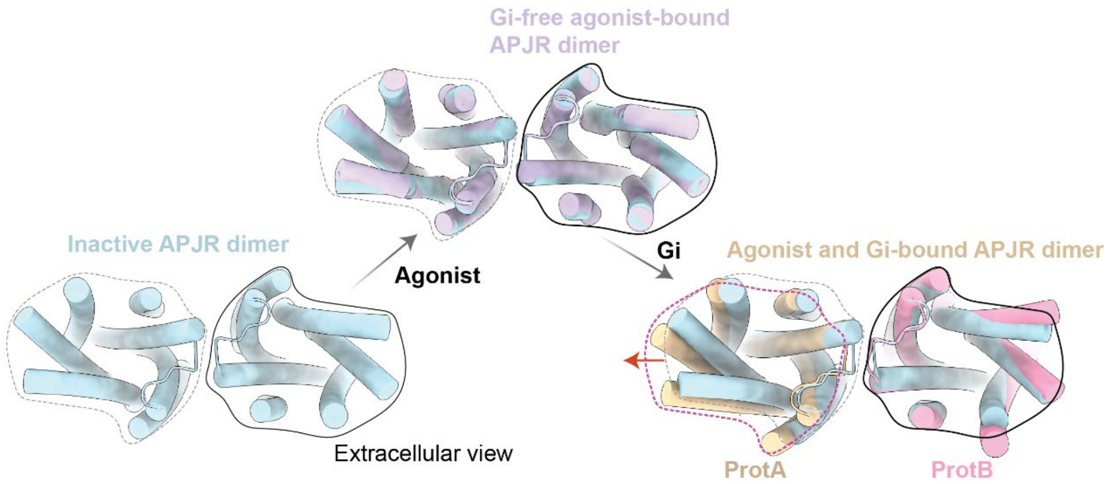

Supplementary Figure 9. The dimerization modes and conformational changes in transmembrane domains of APJR in different functional states. Various dimerization modes of the transmembrane domain of APJR during receptor activation, aligned on ProtBs. The red arrow indicates the movement of ProtA upon agonist and Gi binding.

Supplementary Table 1. Cryo-EM data collection, refinement and validation statistics of G protein coupled GPCR complexes.

	$_{dim}$APJR^{AP13}-Gi	$_{mon}$APJR^{AP13}-Gi	Apo APJR
Data collection and processing			
Magnification	105,000	105,000	105,000
Voltage (kV)	300	300	300
Electron exposure (e-/Å^2)	60	60	60
Defocus range (μm)	-0.7~-2.2	-0.7~-2.2	-0.7~-2.2
images	9108	9108	9108
Pixel size (Å)	1.04	1.04	1.04
Symmetry imposed	C1	C1	C2
Final particles	24,309	40,681	224,827
Map resolution	3.48	3.13	2.97
FSC threshold	0.143	0.143	0.143
Refinement			
Initial model used (PDB code)	7W0L	7W0M	7SUS
Map sharping B factor (Å)	61.0	64.9	96.2
Model composition			
Non-hydrogen atoms	11348	9233	4842
Protein residues	1442	1177	597
Ligand	0	0	2
B-factors			
Protein	90.62	67.71	44.89
Ligand			37.65
R.M.S. deviations			
Bond lengths (Å)	0.003	0.004	0.004
Bond angles (˚)	0.752	0.677	0.664
Validation			
MolProbity score	2.21	2.08	1.89
Clash score	20.32	14.35	12.59
Poor rotamers (%)	0.00	0.00	0.00
Ramachandran plot			
Favored (%)	93.87	93.71	95.94
Allowed (%)	6.13	6.20	4.06
Disallowed (%)	0.00	0.09	0.00
EMD code	38574	38575	38578
PDB code	8XQE	8XQF	8XQI

	JN241-APJR	JN241-9-APJR	JN241-9-APJR-Gi
Magnification	105,000	105,000	105,000
Voltage (kV)	300	300	300
Electron exposure (e-/Å^2)	60	60	60
Defocus range (μm)	-0.7~-2.2	-0.7~-2.2	-0.7~-2.2
images	3115	4,886	4,071
Pixel size (Å)	1.04	1.04	1.04
Symmetry imposed	C2	C1	C1
Final particles	170,746	488,065	65,206
Map resolution	2.95	3.01	3.12
FSC threshold	0.143	0.143	0.143
Refinement			
Initial model used (PDB code)	7SUS	7SUS	7W0L
Map sharping B factor (Å)	85.6	136.8	121.4
Model composition			
Non-hydrogen atoms	6904	6709	10089
Protein residues	864	847	1293
Ligand	4	2	
B-factors			
Protein	58.62	66.42	77.45
Ligand	74.36	49.30	
R.M.S. deviations			
Bond lengths (Å)	0.003	0.003	0.003
Bond angles (˚)	0.568	0.630	0.661
Validation			
MolProbity score	1.83	1.90	2.01
Clash score	10.26	13.38	16.06
Poor rotamers (%)	0.00	0.00	0.00
Ramachandran plot			
Favored (%)	95.68	96.07	95.69
Allowed (%)	4.32	3.93	4.23
Disallowed (%)	0.00	0.00	0.00
EMD code	38579	39810	39816
PDB code	8XQJ	8Z74	8Z7J

Supplementary Table 2. Mutational effects on cAMP accumulation of APJR induced by apelin-13 and ELA measured by Glo-Sensor assays.

Ligands	Apelin-13		ELA		Expression Level±SEM (% WT)
	Gi signaling				
	pEC50±SEM	Emax±SEM (% WT)	pEC50±SEM	Emax±SEM (% WT)	
**WT**	10.68±0.11	100±0.00	10.34±0.09	100±0.00	100±0.00
**F78A**	10.32±0.01^{ns}	83.49±5.84^{ns}	9.40±0.03*	72.46±3.79^{ns}	122.07±13.23^{ns}
**I109A**	10.59±0.06^{ns}	90.86±9.24^{ns}	9.56±0.05^{ns}	86.49±19.63^{ns}	106.40±4.28^{ns}
**M113A**	10.67±0.13^{ns}	79.24±4.12^{ns}	10.49±0.05^{ns}	79.11±1.81^{ns}	84.50±4.41^{ns}
**Y185A**	ND	ND	9.81±0.49^{ns}	50.25±6.43^{ns}	56.82±12.04*
**K268A**	9.39±0.09^{ns}	63.36±7.01^{ns}	8.82±0.23***	25.62±41.00*	124.10±16.95^{ns}
**F291A**	10.66±0.23^{ns}	73.81±12.87^{ns}	10.53±0.10^{ns}	72.46±9.89^{ns}	100.72±18.62^{ns}
**Y299A**	10.28±0.11^{ns}	93.42±10.29^{ns}	10.37±0.27^{ns}	139.30±5.64^{ns}	88.52±1.98^{ns}
**Y271A**	6.07±2.43**	55.76±2.99^{ns}	9.64±0.14^{ns}	80.09±12.24^{ns}	94.03±2.68^{ns}

The data are presented as the mean ± SEM of three independent experiments performed in triplicates (n=3). ND indicates no detectable signal. $^{ns}P > 0.05$, $*P < 0.05$, $**P < 0.01$, $***P < 0.001$ and $****P < 0.0001$ by one-way ANOVA followed by Dunnett's multiple comparisons test, compared with the response of the APJR-WT.

Uncropped SDS PAGE (Supplementary Figure 2d)

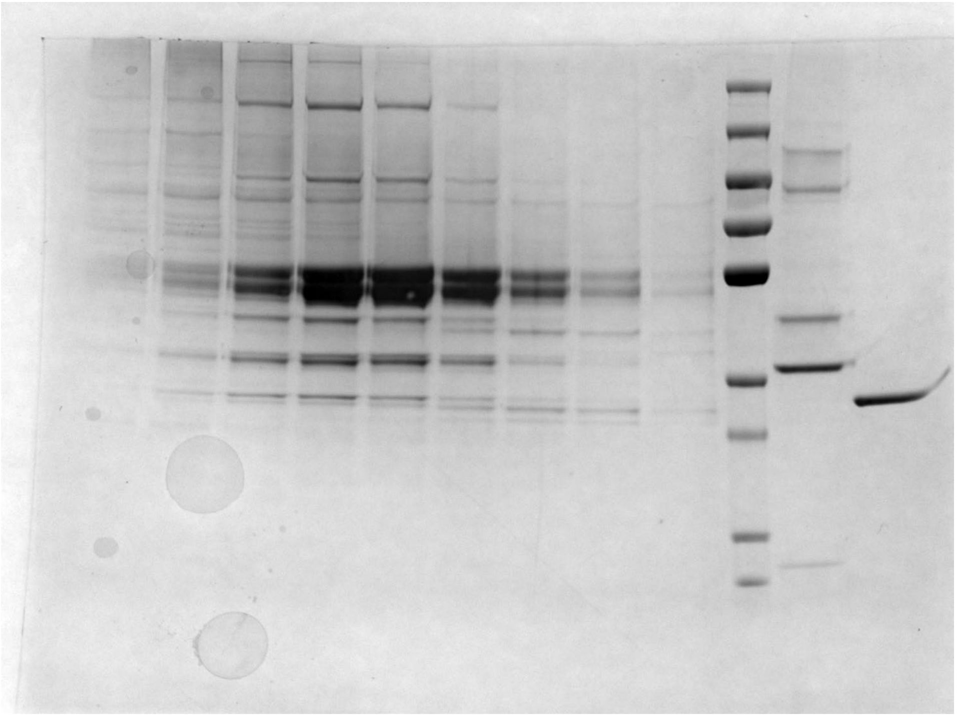

Uncropped SDS PAGE (Supplementary Figure 5d)

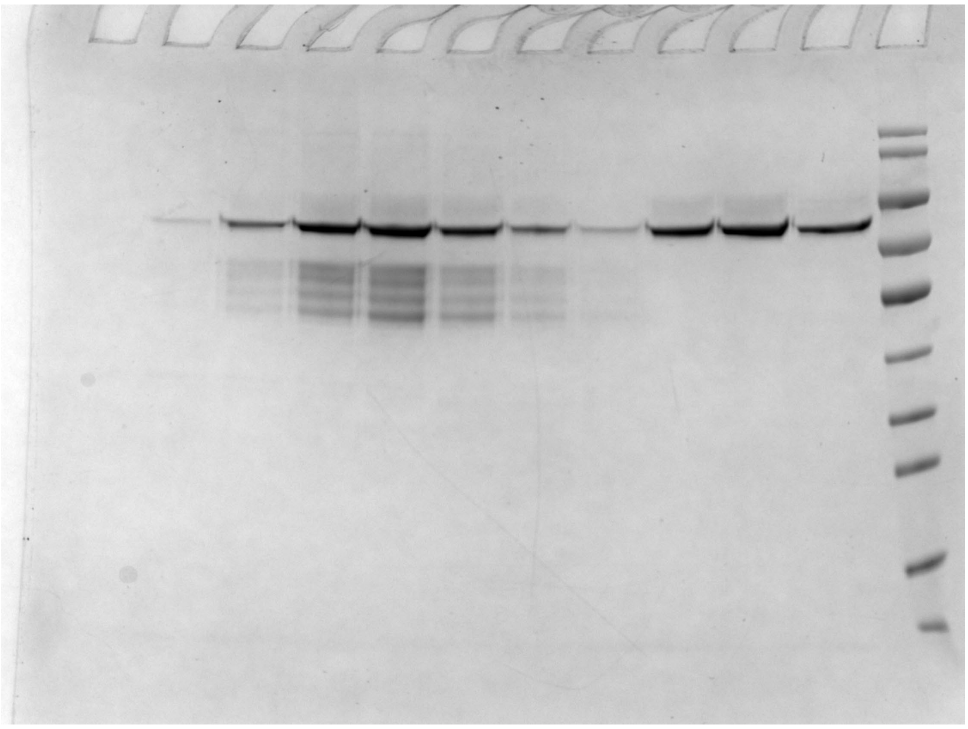

Uncropped SDS PAGE (Supplementary Figure 5h)

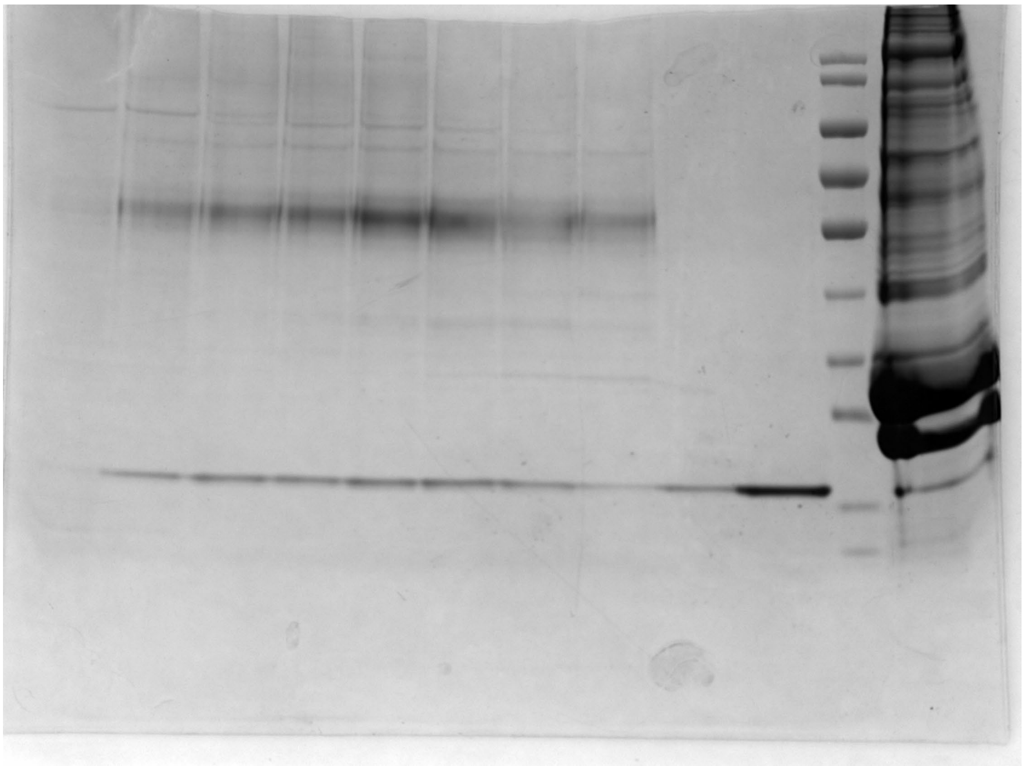

Uncropped SDS PAGE (Supplementary Figure 5i)

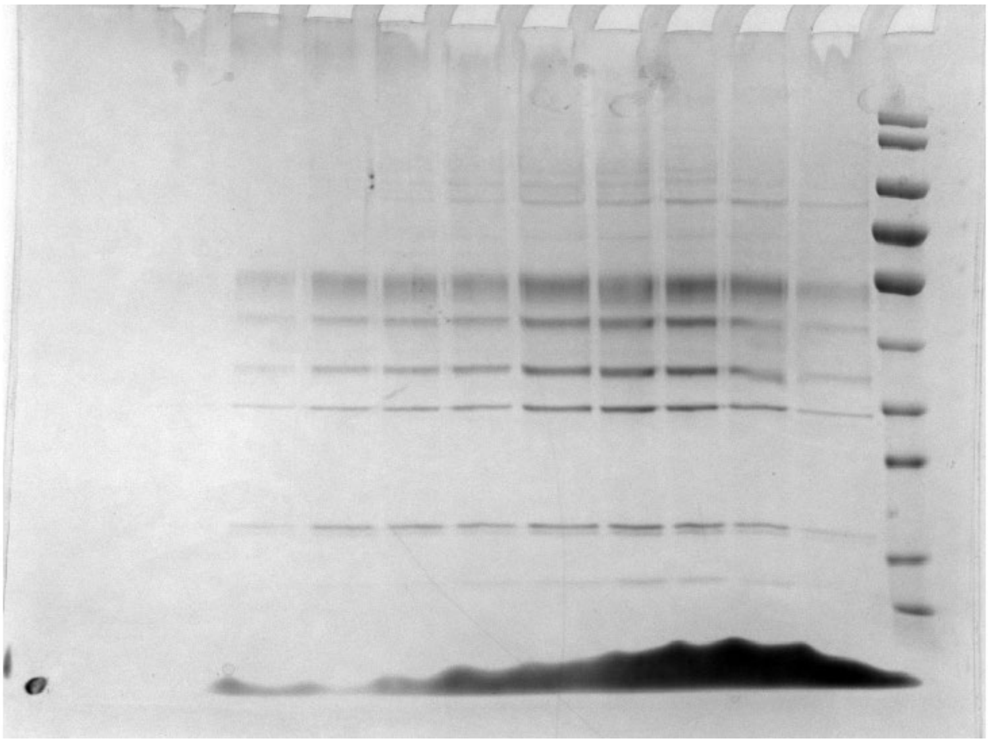

Supplement: Supplementary file 1 — Supplementary Information [file 41467_2024_55555_MOESM1_ESM.pdf]
