# Supplementary material for: Structural insights into the regulation of monomeric and dimeric apelin receptor
Source: Nat Commun. 2025 Jan 2;16:310. doi: 10.1038/s41467-024-55555-6 (PMC11697037; doi:10.1038/s41467-024-55555-6)
Supplement: Supplementary file 2 — Description of Additional Supplementary Files [file 41467_2024_55555_MOESM2_ESM.pdf]

Description of Additional Supplementary Files

File name: Supplementary Data 1
Description: Gromacs *mdp input file for the production run.

File name: Supplementary Data 2
Description: Representative snapshot after 250ns production run for antagonist bound APJR.

File name: Supplementary Data 3
Description: Representative snapshot after 250ns production run for apo APJR.

File name: Supplementary Data 4
Description: Representative snapshot after 250ns production run for AP13 bound APJR.
