# Supplementary material for: Structural insights into the regulation of monomeric and dimeric apelin receptor
Source: Nat Commun. 2025 Jan 2;16:310. doi: 10.1038/s41467-024-55555-6 (PMC11697037; doi:10.1038/s41467-024-55555-6)
Supplement: Supplementary file 7 — Reporting Summary [file 41467_2024_55555_MOESM7_ESM.pdf]

nature portfolio

Corresponding author(s): Fei Xu

Last updated by author(s): Nov 25, 2024

Reporting Summary

Nature Portfolio wishes to improve the reproducibility of the work that we publish. This form provides structure for consistency and transparency in reporting. For further information on Nature Portfolio policies, see our Editorial Policies and the Editorial Policy Checklist.

Statistics

For all statistical analyses, confirm that the following items are present in the figure legend, table legend, main text, or Methods section.

n/a	Confirmed	
☐	☒	The exact sample size (*n*) for each experimental group/condition, given as a discrete number and unit of measurement
☐	☒	A statement on whether measurements were taken from distinct samples or whether the same sample was measured repeatedly
☐	☒	The statistical test(s) used AND whether they are one- or two-sided<br>*Only common tests should be described solely by name; describe more complex techniques in the Methods section.*
☒	☐	A description of all covariates tested
☒	☐	A description of any assumptions or corrections, such as tests of normality and adjustment for multiple comparisons
☐	☒	A full description of the statistical parameters including central tendency (e.g. means) or other basic estimates (e.g. regression coefficient) AND variation (e.g. standard deviation) or associated estimates of uncertainty (e.g. confidence intervals)
☐	☒	For null hypothesis testing, the test statistic (e.g. F, t, r) with confidence intervals, effect sizes, degrees of freedom and P value noted<br>*Give P values as exact values whenever suitable.*
☒	☐	For Bayesian analysis, information on the choice of priors and Markov chain Monte Carlo settings
☒	☐	For hierarchical and complex designs, identification of the appropriate level for tests and full reporting of outcomes
☒	☐	Estimates of effect sizes (e.g. Cohen's *d*, Pearson's *r*), indicating how they were calculated

Our web collection on statistics for biologists contains articles on many of the points above.

Software and code

Policy information about availability of computer code

Data collection	Automated data collections on the Titan Krios were performed using SerialEM v3.8.0.
Data analysis	The following softwares were used in this study: cryoSPARC v.4.3.0, Phenix v.1.20.1, Coot v.0.9.8.5, DeepEMhancer, PyMOL v2.5.8, UCSF Chimera v1.15, UCSF Chimera X v1.7.1, GraphPad Prism 9.0.

For manuscripts utilizing custom algorithms or software that are central to the research but not yet described in published literature, software must be made available to editors and reviewers. We strongly encourage code deposition in a community repository (e.g. GitHub). See the Nature Portfolio guidelines for submitting code & software for further information.

Data

Policy information about availability of data

All manuscripts must include a data availability statement. This statement should provide the following information, where applicable:

- Accession codes, unique identifiers, or web links for publicly available datasets
- A description of any restrictions on data availability
- For clinical datasets or third party data, please ensure that the statement adheres to our policy

The cryo-EM density map generated in this study of the dimAPJRAP13-Gi, monAPJRAP13-Gi, Apo APJR, JN241-APJR, JN241-9-APJR and JN241-9-APJR-Gi have been deposited in the Electron Microscopy Data Bank (EMDB) under accession code EMD-38574 (https://www.ebi.ac.uk/emdb/EMD-38574, dimAPJRAP13-Gi), EMD-38575 (https://www.ebi.ac.uk/emdb/EMD-38575, monAPJRAP13-Gi), EMD-38578(https://www.ebi.ac.uk/emdb/EMD-38578, Apo APJR), EMD-38579 (https://

www.ebi.ac.uk/emdb/EMD-38579, JN241-APJR), EMD-39810 (https://www.ebi.ac.uk/emdb/EMD-39810, JN241-9-APJR), EMD-39816 (https://www.ebi.ac.uk/emdb/EMD-39816, JN241-9-APJR-Gi), and model coordinates have been deposited in the Protein Data Bank (PDB) under accession number 8XQE (https://doi.org/10.2210/pdb8XQE/pdb, dimAPJRAP13-Gi), 8XQF (https://doi.org/10.2210/pdb8XQF/pdb, monAPJRAP13-Gi), 8XQI (https://doi.org/10.2210/pdb8XQI/pdb, Apo APJR), 8XQJ (https://doi.org/10.2210/pdb8XQJ/pdb, JN241-APJR), 8Z74 (https://doi.org/10.2210/pdb8Z74/pdb, JN241-9-APJR) and 8Z7J (https://doi.org/10.2210/pdb8Z7J/pdb, N241-9-APJR-Gi) , respectively. All other data generated in this study are provided in the Supplementary Information/Source Data file.

Research involving human participants, their data, or biological material

Policy information about studies with human participants or human data. See also policy information about sex, gender (identity/presentation), and sexual orientation and race, ethnicity and racism.

Reporting on sex and gender	N/A
Reporting on race, ethnicity, or other socially relevant groupings	N/A
Population characteristics	N/A
Recruitment	N/A
Ethics oversight	N/A

Note that full information on the approval of the study protocol must also be provided in the manuscript.

Field-specific reporting

Please select the one below that is the best fit for your research. If you are not sure, read the appropriate sections before making your selection.

☒ Life sciences ☐ Behavioural & social sciences ☐ Ecological, evolutionary & environmental sciences

For a reference copy of the document with all sections, see nature.com/documents/nr-reporting-summary-flat.pdf

Life sciences study design

All studies must disclose on these points even when the disclosure is negative.

Sample size	For cryo-EM studies, sample size was determined by availability of the microscope time.
Data exclusions	No data were excluded from the analysis.
Replication	All the MD and functional assays were conducted at least three independent experiments with technical repeats. All attempts at replication were successful.
Randomization	Randomization is not relevant to this study, since no experimental group was assigned in all experiments.
Blinding	Blinding is not applicable to this study, since neither structural nor functional experiments included subjective assignments.

Reporting for specific materials, systems and methods

We require information from authors about some types of materials, experimental systems and methods used in many studies. Here, indicate whether each material, system or method listed is relevant to your study. If you are not sure if a list item applies to your research, read the appropriate section before selecting a response.

Materials & experimental systems

n/a	Involved in the study	
☒	☐	Antibodies
☐	☒	Eukaryotic cell lines
☒	☐	Palaeontology and archaeology
☒	☐	Animals and other organisms
☒	☐	Clinical data
☒	☐	Dual use research of concern
☒	☐	Plants

Methods

n/a	Involved in the study	
☒	☐	ChIP-seq
☒	☐	Flow cytometry
☒	☐	MRI-based neuroimaging

Eukaryotic cell lines

Policy information about cell lines and Sex and Gender in Research

Cell line source(s)	Trichuplusia ni Hi5 insect cells (Invitrogen, B85502)
Authentication	Authentication was done by the manufacturers by standard STR profiling analysis.
Mycoplasma contamination	All cell lines tested are negative for mycoplasma contamination.
Commonly misidentified lines (See ICLAC register)	No commonly misidentified cell lines were used.

Plants

Seed stocks	N/A
Novel plant genotypes	N/A
Authentication	N/A
